# Mycophenolate Mofetil-Induced Cytomegalovirus Colitis in a Patient With Polymyositis

**DOI:** 10.7759/cureus.28848

**Published:** 2022-09-06

**Authors:** Navin D Naik, Joy Elliott

**Affiliations:** 1 Gastroenterology, Edward Via College of Osteopathic Medicine, Blacksburg, USA; 2 Family Medicine, Edward Via College of Osteopathic Medicine, Blacksburg, USA

**Keywords:** mycophenolate mofetil, immunosuppression, infectious colitis, persistent diarrhea, cytomegalovirus (cmv)

## Abstract

As the rate of autoimmune conditions and cancers is increasing in the United States, a larger number of patients are being managed with immunosuppressive medications. Diarrhea is a common problem in immunocompromised patients. A feared complication of immunosuppression is infection with opportunistic pathogens. Cytomegalovirus is an opportunistic infection that can cause infection in a variety of different organ systems, including affecting the gastrointestinal system. Severe infection is most commonly seen as a complication of acquired immunodeficiency syndrome (AIDS), organ transplant, hematological malignancy, or cancer therapy. This case report describes a case of cytomegalovirus colitis in an immunosuppressed patient following mycophenolate mofetil (CellCept) therapy.

## Introduction

Autoimmune conditions are increasing in prevalence in the United States [1]. This, combined with the increasing prevalence of cancer, is leading to a greater number of patients taking immunosuppressive medication. Diarrhea is a common problem in immunocompromised patients and can be difficult to manage due to several potential etiologies. Infectious causes from both opportunistic pathogens and other human pathogens must be considered, as well as side effects from medications, dietary problems, and normal disease course [2]. Cytomegalovirus (CMV) infection in the setting of iatrogenic immunosuppression is a dangerous complication of treatment. CMV is a double-stranded DNA virus of the Herpesviridae family. Like other viruses in this family, CMV can remain latent in macrophages after primary infection [3]. Therefore, reactivation of latent infection is a possibility, especially after immune system dysfunction. Primary CMV infection in immunocompetent patients is generally asymptomatic [4]. Symptomatic immunocompetent patients may experience CMV mononucleosis-like syndrome that can last many weeks [5]. In immunocompromised patients, reactivation or primary infection can cause infection in a variety of organ systems. CMV can affect the colon, esophagus, retina, lungs, and brain. It is a frequent complication after organ transplantation [6] and is the most common opportunistic infection in liver transplant patients [7]. CMV infection is also common in patients with advanced HIV disease, with retinitis being the most common presentation [8]. In AIDS patients, CMV infection is generally seen in those with a CD4 count of less than 50. Due to the very effective, highly active anti-retroviral therapies present today, CMV infection in AIDS patients has decreased dramatically in prevalence [9]. In pregnant women, CMV is one of the TORCH (toxoplasmosis, rubella, cytomegalovirus, herpes, and other agents) infections. CMV can cause a variety of symptoms in the unborn fetus and neonate such as hepatomegaly, seizures, periventricular calcifications, mental retardation, sensorineural hearing loss, and hydrops fetalis [10]. First-line treatment for CMV infection requiring intervention is intravenous ganciclovir or oral valganciclovir [11]. Foscarnet may also be used if the patient cannot tolerate ganciclovir or is infected with a strain of CMV that is resistant.

Mycophenolate mofetil (MMF) is an immunosuppressant, primarily used in organ transplant patients to prevent rejection. The literature describes cases of CMV colitis in patients following treatment with MMF for Wegener’s granulomatosis [12] and birdshot chorioretinopathy [13] as well as an increased risk of CMV infection following MMF use for allogenic stem cell transplantation [14] and cadaveric kidney transplantation [15]. MMF has proved efficacious in treating polymyositis but should only be reserved for when standard therapies fail [16].

This paper describes a case of MMF-induced immunosuppression, complicated by CMV colitis, in a patient with a history of polymyositis.

## Case presentation

The patient was a 69-year-old African American with a past medical history of polymyositis, Sjogren’s, chronic kidney disease stage 3, atrial fibrillation not on anticoagulation, and type 2 diabetes mellitus. He presented to the emergency department with a chief complaint of frequent bowel movements and decreased urine output. Bowel movements occurred 30 minutes after every meal for several weeks prior to presentation. The patient described the stools as mostly regular but occasionally loose. The patient was experiencing decreased urine output over several days. There was also decreased oral intake of food and water. Ten months prior to presentation, the patient began having weakness in the upper and lower extremities bilaterally and was sent to a rheumatologist for further workup. Six months later, the patient was diagnosed with polymyositis and was started on prednisone and MMF for the polymyositis treatment and trimethoprim/sulfamethoxazole (TMP/SMX) for infection prophylaxis. At this time, the patient was non-ambulatory and bedbound due to bilateral lower extremity weakness. As a consequence of his immobility, he developed a stage I sacral decubitus ulcer. One month prior to the presentation, the patient was admitted for five days for similar gastrointestinal symptoms. He tested positive for Clostridium (C.) difficile DNA but tested negative for the toxin. He was diagnosed with presumed C. difficile colitis, prescribed vancomycin, and discharged. Following that hospital stay, the patient experienced significant physical decline and a weight loss of 50 pounds.

Medications at presentation in the acute setting consisted of daily baby aspirin, insulin glargine, MMF 1500 mg twice daily, prednisone 60 mg daily, pilocarpine 5 mg daily, and TMP/SMX every Monday, Wednesday, and Friday. The patient has a medication allergy to cephalexin. No relevant family history was provided. On review of systems in the ED, the patient was positive for general weakness/fatigue, decreased urine output, decreased appetite and oral intake, leg weakness, and frequent BMs. The review of systems was otherwise negative, including nausea, vomiting, abdominal pain, fever, chest pain, shortness of breath, myalgias, loss of sensation, dysuria, or blood in stool. Initial vital signs were stable with a temperature of 97.7, heart rate of 86, blood pressure of 107/64, respiratory rate of 18, and oxygen saturation of 100% on room air. The physical examination was positive for 2/5 strength in the bilateral lower extremities. Laboratory testing yielded hemoglobin of 9.2, white blood cell count of 2.4, platelet count of 185, sodium of 137, potassium of 4.8, chloride of 115, bicarbonate of 14, blood urea nitrogen (BUN) of 7.4, and creatinine of 3.3. The urinalysis was negative. A chest X-ray and electrocardiogram were performed, both of which were normal. 

**Figure 1:**
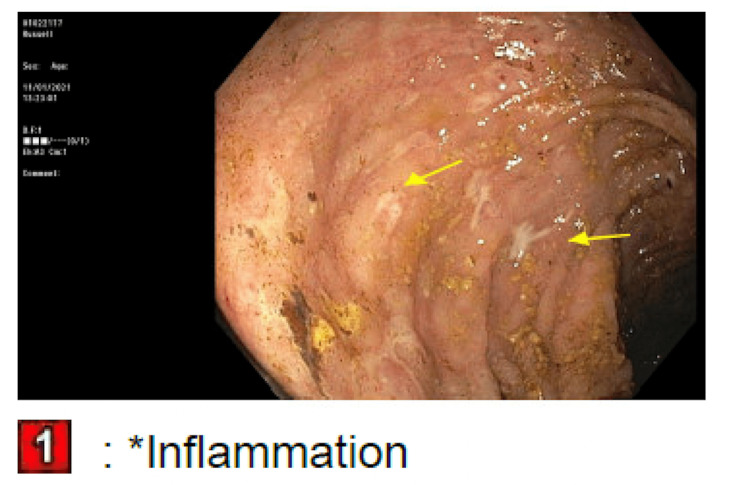
Pictures taken during the patient’s sigmoidoscopy Yellow arrows indicate areas of inflammation and ulceration. 1: rectum

**Figure 2:**
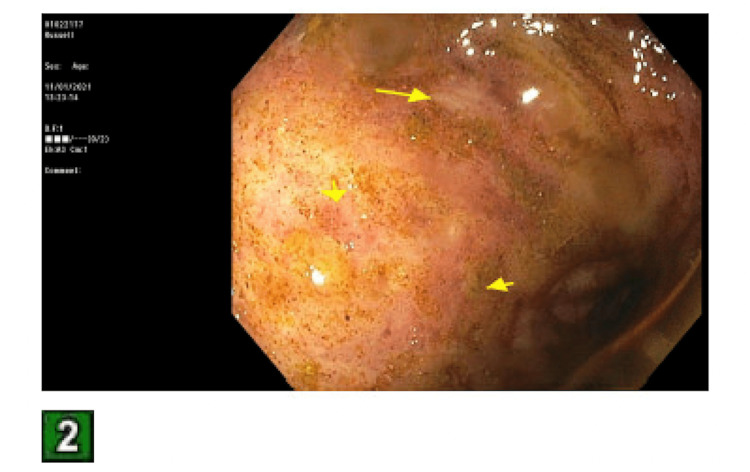
Sigmoid colon

**Figure 3:**
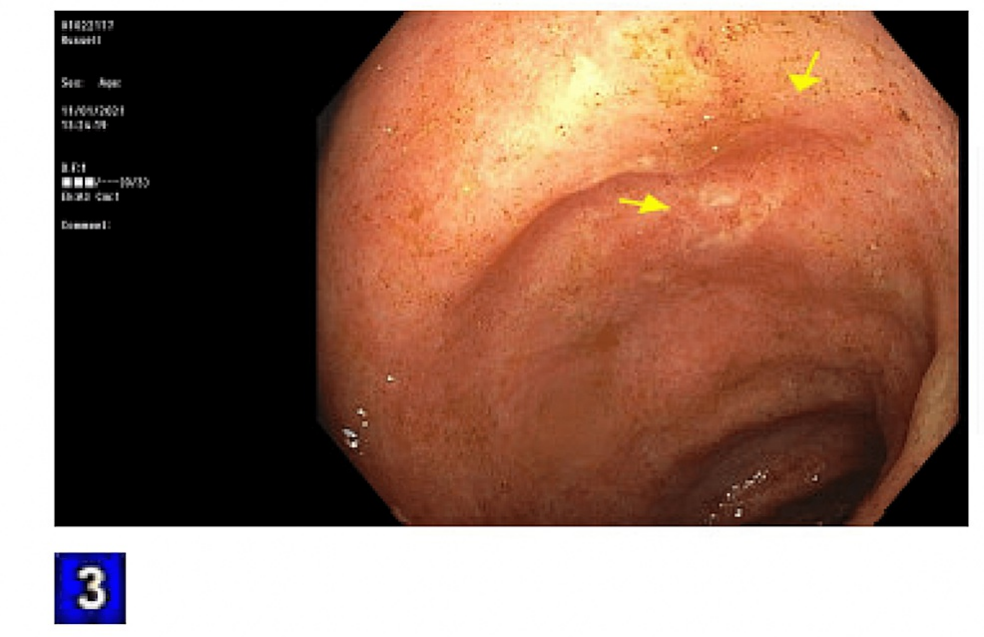
Descending colon

The patient was admitted for an acute kidney injury (AKI) and failure to thrive. The fractional excretion of sodium was calculated to be 0.8%. This, combined with the clinical picture of decreased oral hydration, led to a suspected prerenal etiology for his AKI. Intravenous fluids were started. His stool was tested for C. difficile toxin, which was negative. The day following admission, his frequent BMs began to worsen, and his stool consistency started to become softer and more watery. The TMP/SMX was held, and he was started on loperamide 2 mg as needed. This only provided very minimal relief. Rheumatology was consulted to stop his MMF, and they agreed. Overnight, the patient had a significant drop in hemoglobin to 6.6, and 2 units of packed red blood cells were transfused. The reticulocyte count was 0.6. The stool hemoccult was negative. Additionally, the bicarbonate dropped to 11 overnight and the pH of arterial blood gas was 7.25. Nephrology was consulted, and they started a bicarbonate drip of 150 mEq at 100 mL/hour. At this point, the frequent bowel movements had worsened to diarrhea. The patient was having a watery bowel movement every 30 minutes to an hour. The stool osmolar/osmotic gap was calculated to be 20 mOsm/kg, suggesting secretory diarrhea. The stool was also tested for Shiga toxin 1, Shiga toxin 2, Campylobacter antigen, and Giardia, all of which were negative. Gastroenterology was consulted for diarrhea. They suggested deferring a colonoscopy until the patient had improved his platelet count, which, at this point, had dropped to 30k. Per gastroenterology recommendations, cholestyramine 4 grams twice daily was started. A fecal management system was also put in place due to the patient’s skin breakdown from recurrent diarrhea and subsequent irritation. Hematology was consulted. They offered no interventions other than holding aspirin, as MMF is known to cause myelosuppression. Ciprofloxacin and metronidazole were started empirically. The following day, the patient became anuric and had significant bilateral pitting edema on physical exam. A bladder scan showed 50 milliliters of urine in his bladder. Nephrology was consulted again, and they recommended hemodialysis. The patient also had a witnessed tonic-clonic seizure on this day. Neurology was consulted and levetiracetam was started. A head computerized tomography scan and electroencephalogram were ordered, both of which were normal. Over the next two days, platelets were given. The diarrhea was not improving. Flexible sigmoidoscopy was performed, showing diffuse moderate inflammation characterized by erosions, erythema, and shallow ulcerations in the rectum, sigmoid colon, and descending colon, as seen in Figures 1-3. Biopsies were taken and sent to pathology for evaluation.

The following day, the biopsy results came back positive for CMV infection. A serum CMV level was ordered, which resulted in immunoglobulin G (IgG) levels. Infectious disease was consulted, and they recommended stopping the ciprofloxacin and metronidazole and starting the patient on intravenous ganciclovir. The patient continued to experience tonic-clonic seizures and the levetiracetam dose was increased. An MRI was ordered, which was negative. There was a small suspicion of CMV encephalitis, but a lumbar puncture was not done due to the low platelet count. Two days after initiation of intravenous ganciclovir, diarrhea began to slowly improve. After a week, the patient was having four to five bowel movements a day, and they were more regular and well-formed. Hemoglobin was stable between 8 and 9 and the white blood cell and platelet counts were back to baseline. The patient was discharged from the hospital after a week and transferred to a skilled nursing facility, where he also continued hemodialysis. 

## Discussion

Although the prevalence of CMV is common, symptomatic CMV infection is rare. This patient was HIV-negative and was not the recipient of an organ transplant. The authors theorize that after the immune suppression was caused by the MMF, the patient’s latent CMV was reactivated. It is suspected that the MMF also contributed heavily to his pancytopenia, although the CMV infection probably also played a role.

It is important to have a broad differential when treating immunocompromised patients. Diarrhea is a common complication in this population and is associated with increased morbidity. As diarrhea is a side effect of MMF and TMP/SMX, both of those medications were held. When diarrhea persisted, infectious causes were explored. C. difficile is the most common bacterial pathogen in immunocompromised patients (2). Given the patient’s previous admission for assumed C. difficile colitis, that was the leading differential. When the patient’s C. difficile toxin came back negative, other common bacterial and parasitic toxins were tested. Gastroenterology also suggested the patient might have ulcerative colitis but that was less likely given the non-bloody secretory diarrhea. CMV should have been higher on the differential given the patient’s immune status and that MMF has been associated with an increased risk of CMV infection [13,14].

In regard to his progressive kidney failure, it is suspected the patient was experiencing polymyositis-induced glomerulonephritis, as 10.7% of patients with an inflammatory myopathy will develop an AKI, and 20.7% of patients will develop chronic kidney disease. Patients who underwent a kidney biopsy were found mainly to have immune-complex glomerulonephritis on pathology [17]. The etiology of his seizures was unknown, but CMV encephalitis was lower on the differential, as it normally presents with altered mental status, confusion, reduced concentration, and focal signs rather than seizures [18]. A more likely cause would be uremia caused by his kidney failure. Although he was admitted for his AKI, the CMV infection quickly became his most significant problem.

## Conclusions

Iatrogenic immunosuppression following the treatment of necessary diagnoses is a serious problem that can have severe consequences. Strong immunosuppressive medications warrant a discussion between clinicians and patients. As diarrhea is a very common complaint in an immunocompromised patient, clinicians should be mindful of keeping a broad differential. Clinicians should be cognizant of CMV reactivation and disease, especially when the patient is being treated with MMF, given the potential increased risk of CMV.
